# Development and evaluation of a clinical-biochemical nomogram for differentiating pleomorphic adenoma and Warthin’s tumor in the parotid gland

**DOI:** 10.3389/fonc.2025.1565727

**Published:** 2025-08-29

**Authors:** Xiaoying Huang, Feng Zhao, Junkun He, Jiping Su, Zhe Zhang

**Affiliations:** Department of Otolaryngology, Head and Neck Surgery, The First Affiliated Hospital of Guangxi Medical University, Nanning, China

**Keywords:** pleomorphic adenoma, Warthin’s tumor, differential diagnosis, white blood cell count, metabolic syndrome

## Abstract

**Objectives:**

This study aims to construct and evaluate an integrated nomogram model that combines clinical characteristics and biochemical blood markers to enable accurate and cost-effective differentiation between PA and WT.

**Methods:**

A retrospective analysis was carried out on patients diagnosed with PA and WT at the First Affiliated Hospital of Guangxi Medical University between 2013 and 2022. The participants were randomly allocated to the training set or validation set in a 3:1 ratio. Independent predictors were identified using the Least Absolute Shrinkage and Selection Operator (LASSO) and logistic regression. Based on these predictor variables, a nomogram model was developed. The study systematically evaluated the diagnostic performance, calibration effects, and clinical utility of the model through the application of receiver operating characteristic curves (ROC), calibration curves, decision curve analysis (DCA), and clinical impact curves (CIC).

**Results:**

The study cohort included 249 patients with PA and 173 with WT. The participants were split into a training set of 316 and a validation set of 106. Six independent predictors were identified: sex, age, smoking history, bilateral parotid involvement, white blood cell (WBC) count, and postprandial blood glucose at 2 hours (PBG2h). The AUC of the nomogram on the training set was 0.951, and the sensitivity, specificity and accuracy were 0.91, 0.906 and 0.908, respectively; on the validation set, these metrics were 0.934, 0.918, 0.844 and 0.887, respectively. The calibration curves were in close alignment with the ideal diagonals, indicating that the model has excellent calibration performance. Significant net clinical benefit was observed in the DCA and CIC analyses when the threshold probabilities were in the range of 0.05 to 0.97 for the training set and 0.07 to 0.96 for the validation set.

**Conclusion:**

This study presents the first nomogram model integrating clinical features and biochemical indicators for preoperative differentiation between PA and WT. The model offers an accurate, cost-effective tool to support personalized treatment decisions.

## 1 Introduction

Parotid gland tumors are broadly categorized into benign and malignant lesions, with benign parotid gland tumors accounting for approximately 80%, most frequently observed as pleomorphic adenoma (PA) and Warthin’s tumor (WT) (1). Although these two tumors exhibit similar clinical presentations, their biological differences significantly influence treatment strategies. Pleomorphic adenoma has a reported recurrence rate of 14.1% to 42% at 5 years (2, 3) and 4% to 31.4% at 10 years (3, 4) after surgery, and untreated cases carry a 5.9% risk of malignant transformation, necessitating radical surgical intervention (5). In contrast, Warthin’s tumor, with a malignancy rate of less than 1%, is typically managed through conservative approaches or local excision (6). Recent epidemiological studies have shown an increasing incidence of WT, with it now surpassing PA as the most common benign parotid tumor in certain populations. For example, data from Germany indicate that WT accounted for 42.1% of benign tumors over a 42-year period (7) and increased to 53.4% in a more recent decade-long study (8). This rise may be attributed to factors such as aging populations, chronic inflammation, smoking, obesity, metabolic syndrome, and improvements in imaging techniques (7, 9–11). Thus, accurately differentiating PA from WT before surgery is critical for selecting the appropriate treatment plan and optimizing patient outcomes.

While imaging techniques such as ultrasound, computed tomography (CT), magnetic resonance imaging (MRI), and fine-needle aspiration cytology (FNAC) are often employed for preoperative diagnosis, these methods have limitations. The diagnostic accuracy of FNAC ranges from 72% to 86%, with a higher false-negative rate for WT (12, 13), and may cause complications such as pain, bleeding, infection, or transient facial paralysis (14, 15). Imaging techniques provide basic tumor information but are limited by high costs, technical requirements, and potential radiation risks, particularly in patients with renal insufficiency (16–18). Notably, WT frequently presents with cystic formations on imaging—especially bilateral or multifocal cases—characterized by smooth walls, papillary projections, cholesterol crystals (hyperintense on T1-weighted MRI), and rapid washout on dynamic contrast enhancement (19, 20). However, these features are not pathognomonic and can overlap with other cystic salivary gland lesions. Although radiomics models show promise in differentiating PA from WT, their application remains limited due to small sample sizes, inconsistent feature selection, and the lack of external validation (21).

Research has shown that WT is more common in elderly male smokers (22), and immune-inflammatory markers such as the neutrophil-to-lymphocyte ratio (NLR), platelet-to-lymphocyte ratio (PLR), and systemic immune-inflammation index (SII) can assist in distinguishing benign from malignant parotid tumors (23, 24). Additionally, the development of WT may be associated with metabolic syndrome and obesity (9). However, predictive models based on these markers remain underdeveloped. To address this gap, the present study utilizes a 10-year retrospective cohort from a tertiary hospital in Southern China to analyze the clinical features, sociodemographic data, and preoperative inflammatory, immune, and metabolic indicators of PA and WT patients. A simple, accurate diagnostic model is constructed to provide a low-cost, non-invasive, and efficient preoperative diagnostic tool aimed at optimizing treatment strategies and improving patient prognosis.

## 2 Materials and methods

### 2.1 Research objects

**Figure 1:**
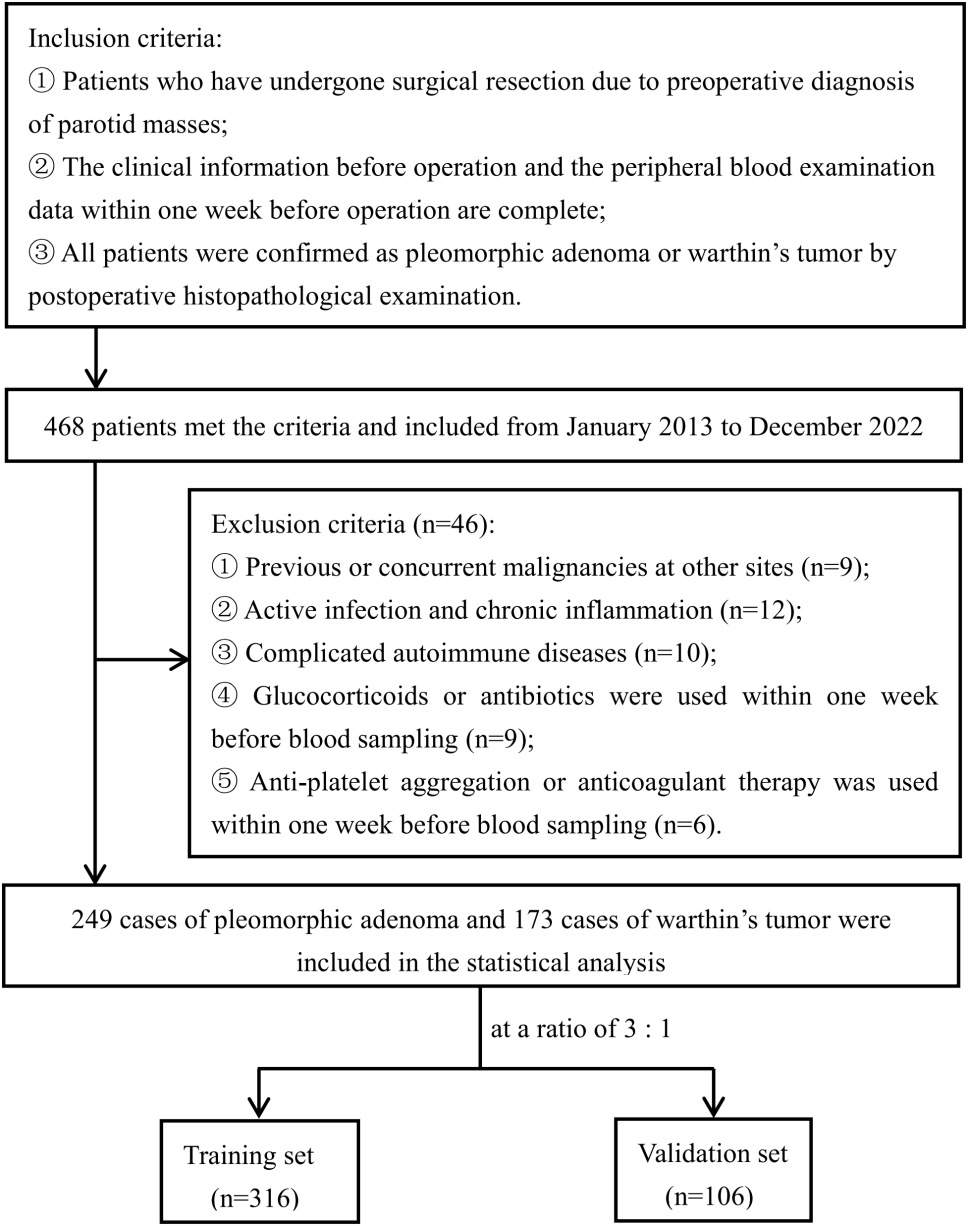
Flowchart of the study design.

This study adheres to the TRIPOD guidelines (25), and data were collected from patients who underwent parotid tumor surgery in the Department of Otorhinolaryngology-Head and Neck Surgery at the First Affiliated Hospital of Guangxi Medical University between January 2013 and December 2022. The inclusion criteria were defined as: preoperative diagnosis of a parotid mass and subsequent surgical resection; availability of preoperative clinical data, peripheral blood biochemical test results within one week before surgery, and complete imaging records (MRI or CT); and postoperative pathological diagnosis of PA or WT. The exclusion criteria included malignant tumors from other locations, active infections or chronic inflammation, autoimmune diseases, or the use of corticosteroids or antibiotics within one week before surgery. The Institutional Review Board approved the study (No. 2023-E339-01), and each patient provided informed consent. The research process is shown in **Figure 1**.

### 2.2 Data collection

One week before surgery, preoperative peripheral blood tests (including complete blood count, liver function, renal function, and blood glucose) were performed, and sociodemographic data, clinical symptoms, signs, and other relevant information were gathered from the hospital’s computerized medical records. Potential biomarkers were calculated, including body mass index (BMI), NLR, PLR, SII, and lymphocyte-to-monocyte ratio (LMR). Specific parameters are listed in **Table 1**. The largest tumor diameter was measured from CT or MRI images. Patients were categorized into married and unmarried groups (including divorced individuals) based on their marital status. Smoking was defined as having smoked continuously for at least six months prior to surgery, with a daily intake of more than one cigarette. Drinking was defined as consuming alcohol continuously for at least six months prior to surgery, with a weekly alcohol intake of 72 grams or more. A BMI of ≥25.0 kg/m² was considered overweight. Fasting blood glucose (FPG) was measured from venous blood samples taken before breakfast, and PBG2h was measured two hours after breakfast. Other blood samples were taken from 6 to 8 in the morning. The blood samples were analyzed in the hospital lab.

**Table 1:** Baseline characteristics of patients with WT and PA in the total dataset.

Variables	Overall (N=422)	WT (n=173)	PA (n=249)	P-value
Sex, n(%)				
Male	268 (63.51)	165 (95.38)	103 (41.37)	<0.001
Female	154 (36.49)	8 (4.62)	146 (58.63)	
Marital status, n(%)				
Unmarried	70 (16.59)	5 (2.89)	65 (26.10)	<0.001
Married	352 (83.41)	168 (97.11)	184 (73.90)	
Smoking history, n(%)				
NO	235 (55.69)	37 (21.39)	198 (79.52)	<0.001
YES	187 (44.31)	136 (78.61)	51 (20.48)	
Drinking status, n(%)				
NO	308 (72.99)	93 (53.76)	215 (86.35)	<0.001
YES	114 (27.01)	80 (46.24)	34 (13.65)	
History of hypertension, n(%)				
NO	340 (80.57)	115 (66.47)	225 (90.36)	<0.001
YES	82 (19.43)	58 (33.53)	24 (9.64)	
History of diabetes, n(%)				
NO	404 (95.73)	163 (94.22)	241 (96.79)	0.199
YES	18 (4.27)	10 (5.78)	8 (3.21)	
Pain/tenderness, n(%)				
NO	371 (87.91)	148 (85.55)	223 (89.56)	0.214
YES	51(12.09)	25 (14.45)	26 (10.44)	
Parotid involvement, n(%)				
Unilateral	403 (95.50)	155 (89.60)	248 (99.60)	<0.001
Bilateral	19 (4.50)	18 (10.40)	1 (0.40)	
BMI, n(%)				
<25.0 kg/m2	318 (75.36)	118 (68.21)	200 (80.32)	0.005
≥25.0 kg/m2	104 (24.64)	55 (31.79)	49 (19.68)	
Age,year	50.00 (35.00,59.00)	58.00 (52.00,66.00)	38.00 (30.00,50.00)	<0.001
Course of disease,month	12.00 (6.00,48.00)	12.00 (2.00,36.00)	24.00 (8.00,60.00)	<0.001
Maximum tumor diameter,cm	2.70 (2.10,3.50)	3.00 (2.20,3.60)	2.60 (2.00,3.30)	0.001
WBC,×10^9^/L	6.84 (5.67,8.00)	7.41 (6.04,8.65)	6.45 (5.48,7.35)	<0.001
RBC,×10^12^/L	4.72 (4.31,5.12)	4.86 (4.49,5.18)	4.57 (4.22,5.00)	<0.001
HGB,g/L	135.90 (125.90,148.00)	140.30 (131.00,151.00)	132.00 (123.60,144.00)	<0.001
PLT,×10^9^/L	242.60 (201.10,280.70)	240.00 (200.70,281.00)	243.00 (204.00,280.00)	0.645
NEU^a^,×10^9^/L	3.62 (2.86,4.54)	3.93 (3.13,5.03)	3.45 (2.67,4.21)	<0.001
LYM^a^,×10^9^/L	2.21 (1.78,2.74)	2.23 (1.77,2.93)	2.20 (1.78,2.64)	0.254
MONO^a^,×10^9^/L	0.53 (0.43,0.67)	0.58 (0.48,0.74)	0.49 (0.40,0.62)	<0.001
EOS^a^,×10^9^/L	0.20 (0.13,0.34)	0.26 (0.17,0.38)	0.17 (0.10,0.27)	<0.001
BASO^a^,×10^9^/L	0.04 (0.03,0.05)	0.04 (0.03,0.06)	0.03 (0.02,0.05)	<0.001
MPV,fL	8.65 (7.90,9.72)	8.63 (7.76,9.80)	8.68 (7.96,9.70)	0.672
MCV,fL	89.30 (85.43,92.70)	90.65 (85.30,93.60)	88.42 (85.50,92.10)	0.011
MCH,pg	29.80 (28.21,31.00)	30.22 (28.33,31.40)	29.51 (28.21,30.60)	0.01
MCHC,g/L	332.00 (325.70,337.50)	331.80 (324.10,337.60)	332.00 (326.00,337.00)	0.629
RDWCV	0.13 (0.13,0.14)	0.14 (0.13,0.14)	0.13 (0.13,0.14)	<0.001
PCT	0.210 (0.180,0.250)	0.210 (0.180,0.250)	0.210 (0.180,0.250)	0.622
PDW	0.16 (0.13,0.16)	0.16 (0.12,0.16)	0.16 (0.15,0.16)	0.263
HCT	0.410 (0.380,0.440)	0.430 (0.400,0.460)	0.400 (0.370,0.430)	<0.001
FPG,mmol/L	4.61 (4.30,5.05)	4.69 (4.39,5.29)	4.56 (4.23,4.86)	0.001
PBG2h,mmol/L	5.63 (4.88,6.77)	6.34 (5.41,7.56)	5.33 (4.69,6.21)	<0.001
NLR	1.62 (1.28,2.12)	1.71 (1.32,2.34)	1.55 (1.23,1.97)	0.004
PLR	108.08 (86.88,135.35)	106.19 (85.44,130.25)	109.75 (87.81,138.18)	0.224
LMR	4.17 (3.34,5.26)	3.85 (3.03,4.76)	4.35 (3.57,5.56)	<0.001
SII	389.11 (285.51,540.63)	419.17 (307.25,564.23)	377.81 (272.85,514.37)	0.031

^a^Denotes absolute value. Data are presented as median (25% percentile, 75% percentile) for continuous variables and count (percentage) for categorical variables. BMI, body mass index; WBC, white blood cell count; RBC, Red blood cell count; HGB, Hemoglobin; PLT, platelet count; NEU, Neutrophil count; LYM, Lymphocyte count; MONO, Monocyte count; EOS, eosinophil count; BASO, Basophil count; MPV, mean platelet volume; MCV, Mean corpuscular volume; MCH, Mean corpuscular hemoglobin; MCHC, Mean cell hemoglobin concentration; RDWCV, Red blood cell distribution width-coefficient of variation; PCT, plateletcrit; PDW, platelet volume distribution width; HCT, hematocrit; FPG, fasting blood glucose; PBG2h, 2h postprandial blood glucose; NLR, neutrophil-to-lymphocyte ratio; PLR, platelet-to-lymphocyte ratio; LMR, lymphocyte-to-monocyte Ratio; SII, the systemic immune-inflammation index=PLT×NEU/LYM.

### 2.3 Model construction and statistical analysis

Continuous variables with outliers (defined as deviations from the mean by two standard deviations or more) and a small number of missing values were addressed using median interpolation. The chi-squared test was used to evaluate differences in categorical variables between groups. For variables with a normal distribution, the mean ± standard deviation was employed as a means of presenting the data, with the use of an independent samples t-test. On the other hand, for non-normally distributed variables, where the data were represented using medians and interquartile ranges, the appropriate statistical test was the Mann-Whitney U test.

Participants were randomly assigned to the training and validation sets in a 3:1 ratio, and the balance of the datasets was assessed. A LASSO regression method based on ten-fold cross-validation was used in the feature selection process with the parameter λ=100. Stepwise backward logistic regression was employed to identify independent factors and build the model. The discriminative ability of the model was evaluated using the ROC, with the optimal cut-off value established by the maximum Youden index. This was followed by the calculation of the AUC, sensitivity, specificity, and accuracy. Differences in AUC values were compared using the DeLong test. Bootstrap resampling (boot = 500) was applied to obtain confidence intervals for the AUC values and to generate calibration curves for both datasets. The goodness of fit of the model was assessed using the Hosmer-Lemeshow test. Additionally, DCA and CIC were used to evaluate the applicability and net benefit of the model in clinical practice. All statistical analyses were performed using SPSS 23.0 and R 4.2.3 software, with the significance level set at p < 0.05.

## 3 Results

### 3.1 Baseline characteristics

The research sample comprised 249 patients with PA (excluding 26 cases) and 173 patients with WT (excluding 20 cases), resulting in a PA-to-WT ratio of 1.44:1. With the exception of nine additional markers, including pain and soreness, and the history of diabetes, all other characteristics exhibited significant differences when the two groups of individuals were compared (p < 0.05, **Table 1**).

### 3.2 Model development

**Figure 2:**
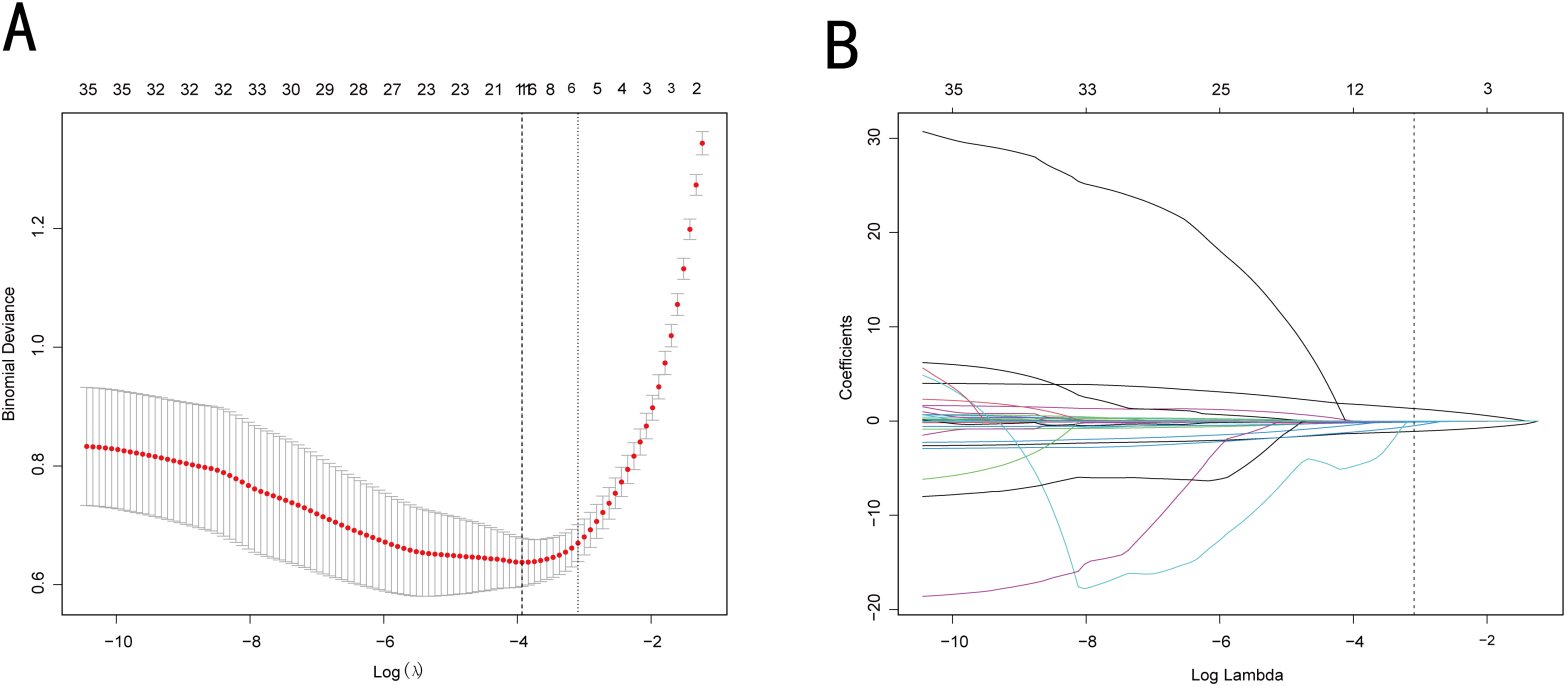
LASSO regression analysis was used for feature selection. **(A)** As λ of the LASSO algorithm changed, the LASSO coefficient profiles of the 35 features in the group were observed. **(B)** The optimal penalty coefficient lambda (λ) was identified in the LASSO model, and a 10-fold cross-validation was carried out in the group.

**Figure 3:**
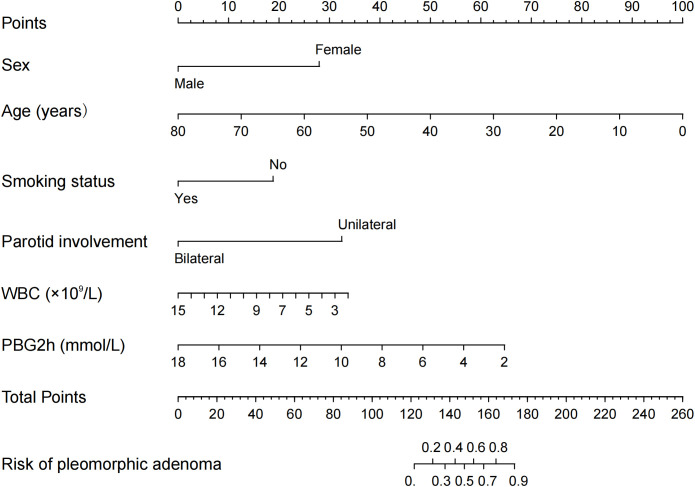
The nomogram for differential diagnosis of PA and WT.

In accordance with the 3:1 random allocation principle, 316 patients were allocated to the training dataset, while the remaining 106 patients were assigned to the validation dataset. No notable differences were observed between the sets regarding any of the variables (all, p > 0.05; **Table 2**). Using the LASSO method, six potential predictors were identified: gender, age, smoking history, bilateral parotid involvement, WBC, and PBG2h (**Figure 2**). These variables were confirmed as independent predictors through logistic regression (**Table 3**; **Figure 3**).

**Table 2:** Baseline characteristics of individuals in training set and validation set.

Variables	Training set (n=316)	Validation set (n=106)	P value
Tumor, n(%)			
WT	128 (40.51)	45 (42.45)	0.724
PA	188 (59.49)	61 (57.55)	
Sex, n(%)			
Male	196 (62.03)	72 (67.92)	0.275
Female	120 (37.97)	34 (32.08)	
Marital status, n(%)			
Umarried	52 (16.46)	18 (16.98)	0.9
Married	264 (83.54)	88 (83.02)	
Smoking history, n(%)			
NO	177 (56.01)	58 (54.72)	0.816
YES	139 (43.99)	48 (45.28)	
Drinking status, n(%)			
NO	237 (75.00)	71 (66.98)	0.108
YES	79 (25.00)	35 (33.02)	
History of hypertension, n(%)			
NO	253 (80.06)	87 (82.08)	0.65
YES	63 (19.94)	19 (17.92)	
History of diabetes, n(%)			
NO	303 (95.89)	101 (95.28)	0.79
YES	13 (4.11)	5 (4.72)	
Pain/tenderness, n(%)			
NO	279 (88.29)	92 (86.79)	0.682
YES	37 (11.71)	14 (13.21)	
Parotid involvement, n(%)			
Unilateral	302 (95.57)	101 (95.28)	0.902
Bilateral	14 (4.43)	5 (4.72)	
BMI, n(%)			
<25.0 kg/m2	234 (74.05)	84 (79.25)	0.283
≥25.0 kg/m2	82 (25.95)	22 (20.75)	
Age,year	50.00 (35.00,59.00)	49.00 (34.00,58.00)	0.793
Course of disease,month	12.00 (6.00,48.00)	13.00 (6.00,60.00)	0.371
Maximum tumor diameter,cm	2.70 (2.10,3.40)	2.80 (2.10,3.60)	0.382
WBC,×10^9^/L	6.87 (5.68,8.00)	6.70 (5.67,7.80)	0.684
RBC,×10^12^/L	4.67 (4.28,5.09)	4.74 (4.38,5.18)	0.216
HGB,g/L	136.00(125.30,149.00)	135.70(129.00,146.00)	0.901
PLT,×10^9^/L	238.90(201.10,279.20)	250.40(204.00,284.40)	0.365
NEU^a^,×10^9^/L	3.64 (2.88,4.57)	3.52 (2.81,4.46)	0.598
LYM^a^,×10^9^/L	2.20 (1.76,2.70)	2.23 (1.82,2.85)	0.345
MONO^a^,×10^9^/L	0.52 (0.42,0.66)	0.53 (0.44,0.68)	0.653
EOS^a^,×10^9^/L	0.21 (0.13,0.34)	0.20 (0.12,0.34)	0.642
BASO^a^,×10^9^/L	0.04 (0.03,0.05)	0.04 (0.03,0.05)	0.626
MPV,fL	8.63 (7.88,9.77)	8.85 (7.96,9.70)	0.503
MCV,fL	89.27 (85.60,92.60)	89.44 (84.20,92.80)	0.898
MCH,pg	29.80 (28.27,31.00)	29.79 (28.00,31.10)	0.997
MCHC,g/L	332.00 (325.00,337.00)	332.00 (326.10,337.60)	0.916
RDWCV	0.13 (0.13,0.14)	0.13 (0.13,0.14)	0.756
PCT	0.210 (0.180,0.250)	0.210 (0.180,0.250)	0.252
PDW	0.16 (0.13,0.16)	0.16 (0.14,0.16)	0.8
HCT	0.410 (0.380,0.442)	0.410 (0.390,0.430)	0.961
FPG,mmol/L	4.66 (4.30,5.06)	4.50 (4.32,4.85)	0.132
PBG2h,mmol/L	5.63 (4.88,6.81)	5.71 (4.92,6.66)	0.606
NLR,median(IQR)	1.65 (1.29,2.15)	1.58 (1.24,2.05)	0.256
PLR,median(IQR)	108.80 (87.63,135.31)	106.74 (84.15,135.35)	0.781
LMR,median(IQR)	4.17 (3.34,5.26)	4.24 (3.45,5.26)	0.661
SII,median(IQR)	391.92 (287.57,553.23)	384.79 (280.90,508.42)	0.648

^a^Denotes absolute value. Data are presented as median (25% percentile, 75% percentile) for continuous variables and count (percentage) for categorical variables. BMI, body mass index; WBC, white blood cell count; RBC, Red blood cell count; HGB, Hemoglobin; PLT, platelet count; NEU, Neutrophil count; LYM, Lymphocyte count; MONO, Monocyte count; EOS, eosinophil count; BASO, Basophil count; MPV, mean platelet volume; MCV, Mean corpuscular volume; MCH, Mean corpuscular hemoglobin; MCHC, Mean cell hemoglobin concentration; RDWCV, Red blood cell distribution width-coefficient of variation; PCT, plateletcrit; PDW, platelet volume distribution width; HCT, hematocrit; FPG, fasting blood glucose; PBG2h, 2h postprandial blood glucose; NLR, neutrophil-to-lymphocyte ratio; PLR, platelet-to-lymphocyte ratio; LMR, lymphocyte-to-monocyte Ratio; SII, the systemic immune-inflammation index=PLT×NEU/LYM.

**Table 3:** Logistic regression analysis for the risk of PA.

Predictor	Estimate	SE	Z	P-value	OR (95%CI)
(Intercept)	9.814	1.552	6.325	<0.001	
Age	-0.106	0.017	-6.408	<0.001	0.899 (0.868–0.927)
WBC	-0.22	0.112	-1.967	0.049	0.802 (0.641–0.996)
PBG2h	-0.344	0.13	-2.65	0.008	0.709 (0.541–0.901)
Female	2.378	0.639	3.722	<0.001	10.78 (3.309–41.866)
Smoking	-1.601	0.452	-3.542	<0.001	0.202 (0.081–0.481)
Bilateral lesion	-2.759	1.349	-2.045	0.041	0.063 (0.002–0.568)

SE, standard error; OR, odds ratio; CI, confidence interval.

### 3.3 Model evaluation

#### 3.3.1 Discriminative ability

**Figure 4:**
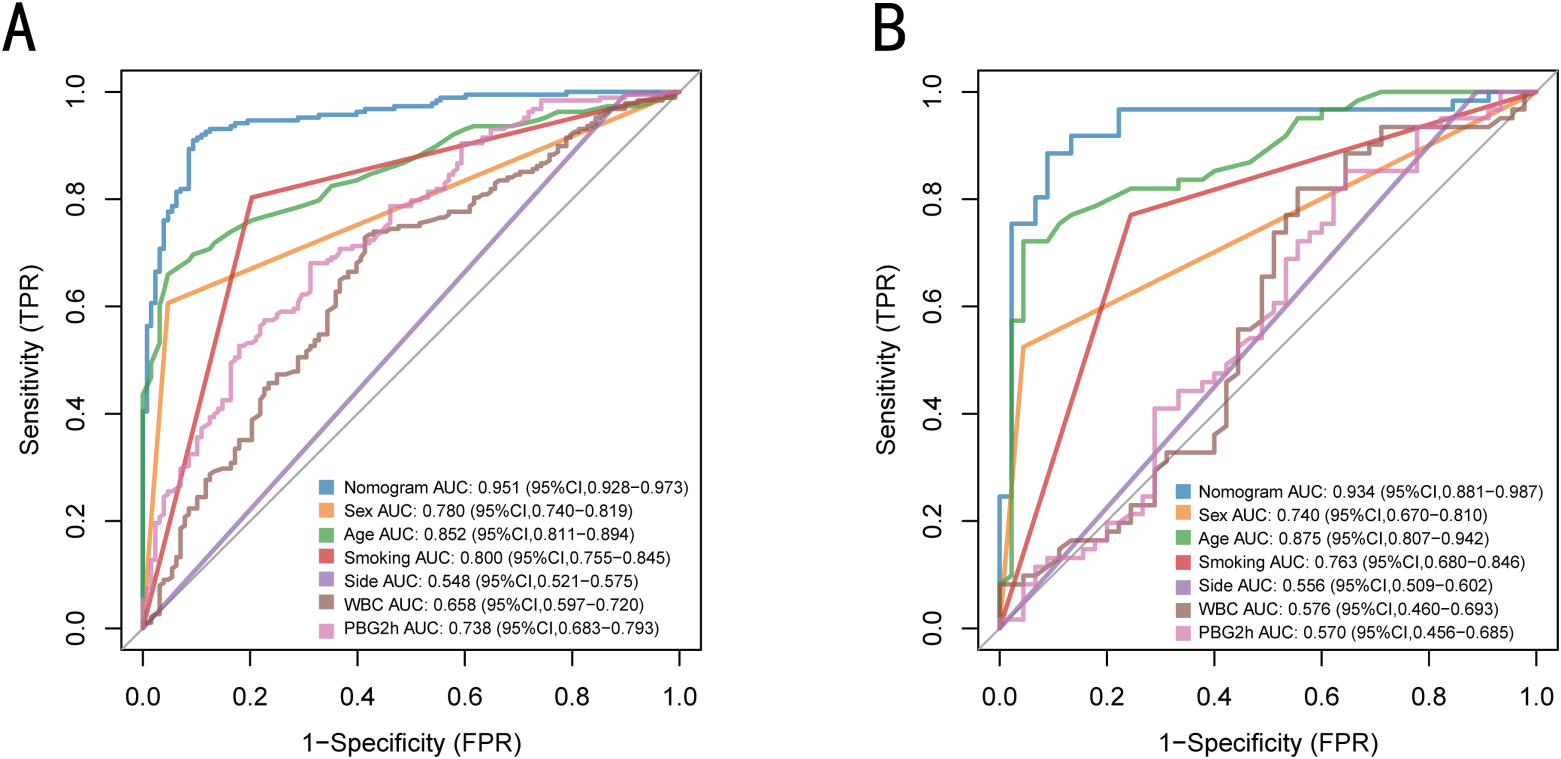
Comparison of ROC curves of the nomogram and six independent predictors in the training set **(A)** and validation set **(B)**.

The nomogram model, based on six variables, demonstrated an AUC of 0.951 (95% confidence interval [CI]: 0.928-0.973) in the training set, and an AUC value of 0.934 (95% CI: 0.881-0.987) in the validation set. The Delong test showed that the discriminative ability of the model was significantly better than that of a single predictor variable (p < 0.05; **Figure 4**; **Table 4**).

**Table 4:** Comparison of the diagnostic efficacy of the nomogram with six independent predictors.

data sets	models	AUC(95%CI)	threshold	specificity	sensitivity	accuracy	p-value
training set	nomogram	0.951 (0.928−0.973)	0.541	0.906 (116/128)	0.91 (171/188)	0.908 (287/316)	–
	Sex	0.780 (0.740−0.819)	0.664	0.953 (122/128)	0.606 (114/188)	0.747 (236/316)	< 0.001
	Age	0.852 (0.811−0.894)	0.74	0.953 (122/128)	0.66 (124/188)	0.778 (246/316)	< 0.001
	Smoking	0.800 (0.755−0.845)	0.56	0.797 (102/128)	0.803 (151/188)	0.801 (253/316)	< 0.001
	Lesion side	0.548 (0.521−0.575)	0.345	0.102 (13/128)	0.995 (187/188)	0.633 (200/316)	< 0.001
	WBC	0.658 (0.597−0.720)	0.582	0.586 (75/128)	0.729 (137/188)	0.671 (212/316)	< 0.001
	PBG2h	0.738 (0.683−0.793)	0.653	0.688 (88/128)	0.681 (128/188)	0.684 (216/316)	< 0.001
validation set	nomogram	0.934 (0.881−0.987)	0.541	0.844 (38/45)	0.918 (56/61)	0.887 (94/106)	–
	Sex	0.740 (0.670−0.810)	0.664	0.956 (43/45)	0.525 (32/61)	0.708 (75/106)	< 0.001
	Age	0.875 (0.807−0.942)	0.74	0.956 (43/45)	0.689 (42/61)	0.802 (85/106)	0.0474
	Smoking	0.763 (0.680−0.846)	0.56	0.756 (34/45)	0.77 (47/61)	0.764 (81/106)	< 0.001
	Lesion side	0.556 (0.509−0.602)	0.345	0.111 (5/45)	1 (61/61)	0.623 (66/106)	< 0.001
	WBC	0.576 (0.460−0.693)	0.582	0.467 (21/45)	0.738 (45/61)	0.623 (66/106)	< 0.001
	PBG2h	0.570 (0.456−0.685)	0.653	0.556 (25/45)	0.525 (32/61)	0.538 (57/106)	< 0.001

The P-value represents whether the ROC curve of independent risk factors is statistically significant compared to the ROC curve of Nomogram by the Delong test.

#### 3.3.2 Calibration

**Figure 5:**
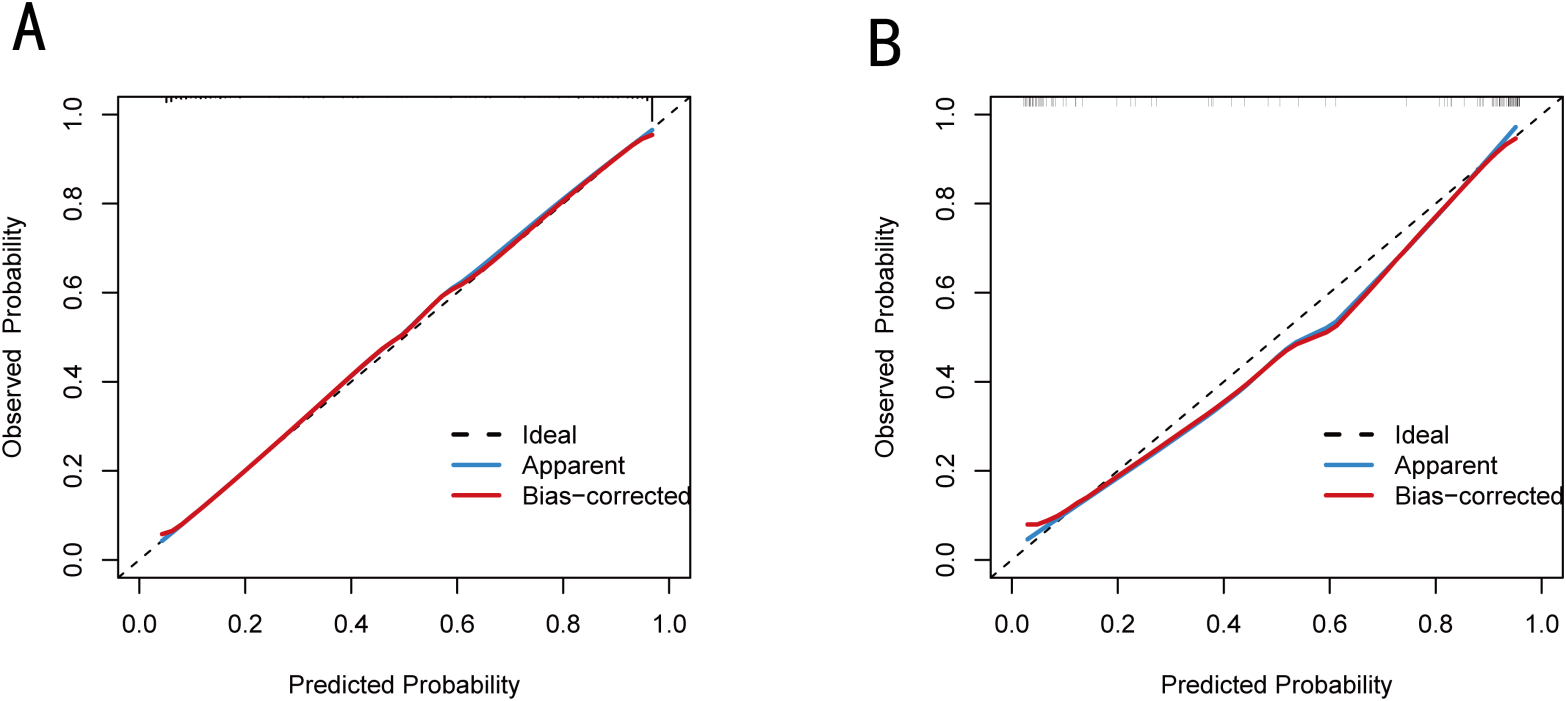
Calibration curves of nomogram in the training set **(A)** and validation set **(B)**.

In the training set, the calibration curve showed an excellent degree of consistency between predictive and actual results, as well as in the validation set (**Figure 5**). The Hosmer-Lemeshow test yielded the χ² value for the training set as 9.492 (p = 0.303) and the χ² value for the validation set as 15.429 (p = 0.051), indicating that the model has good calibration and is suitable for clinical use.

#### 3.3.3 Clinical applicability

**Figure 6:**
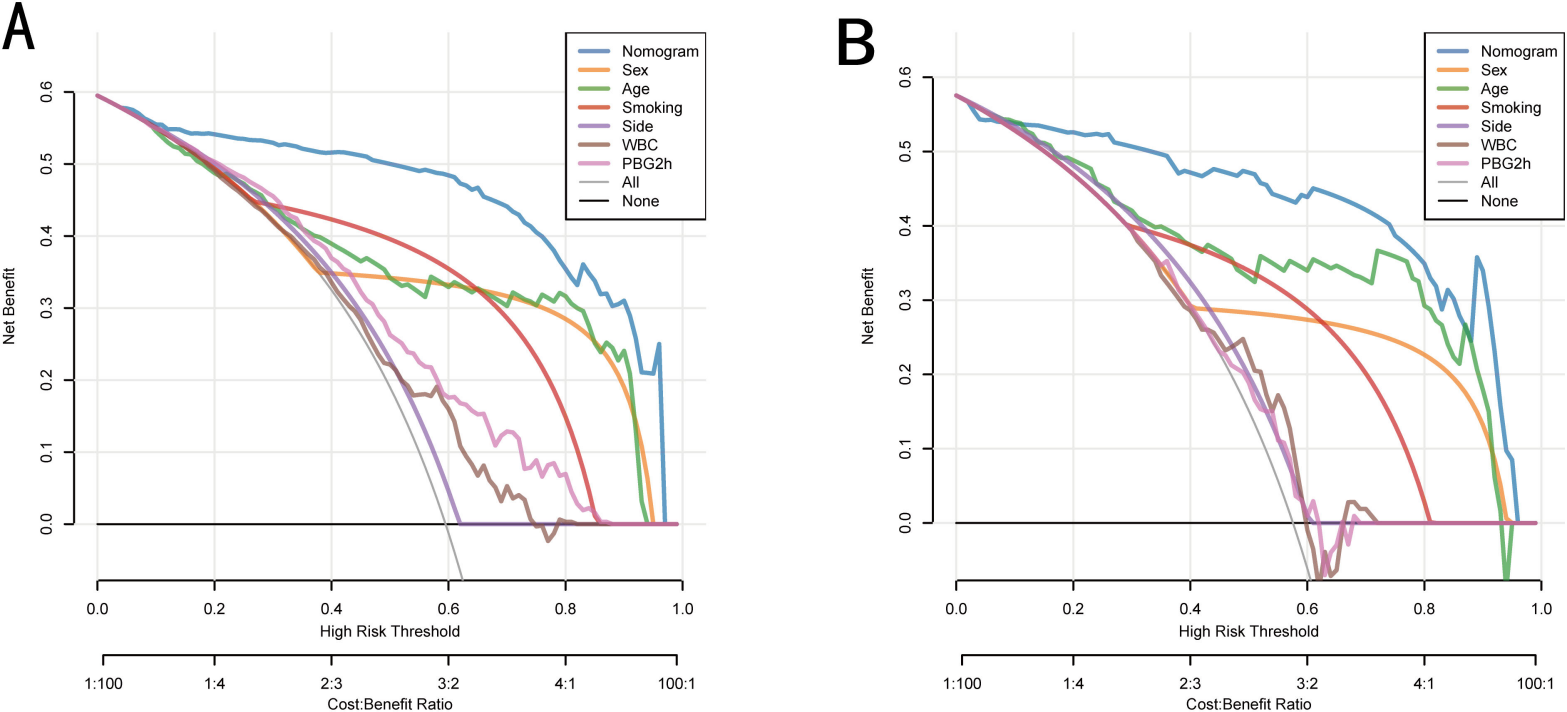
Comparison of DCA of the nomogram with 6 independent risk factors in the training set **(A)** and validation set **(B)**.

**Figure 7:**
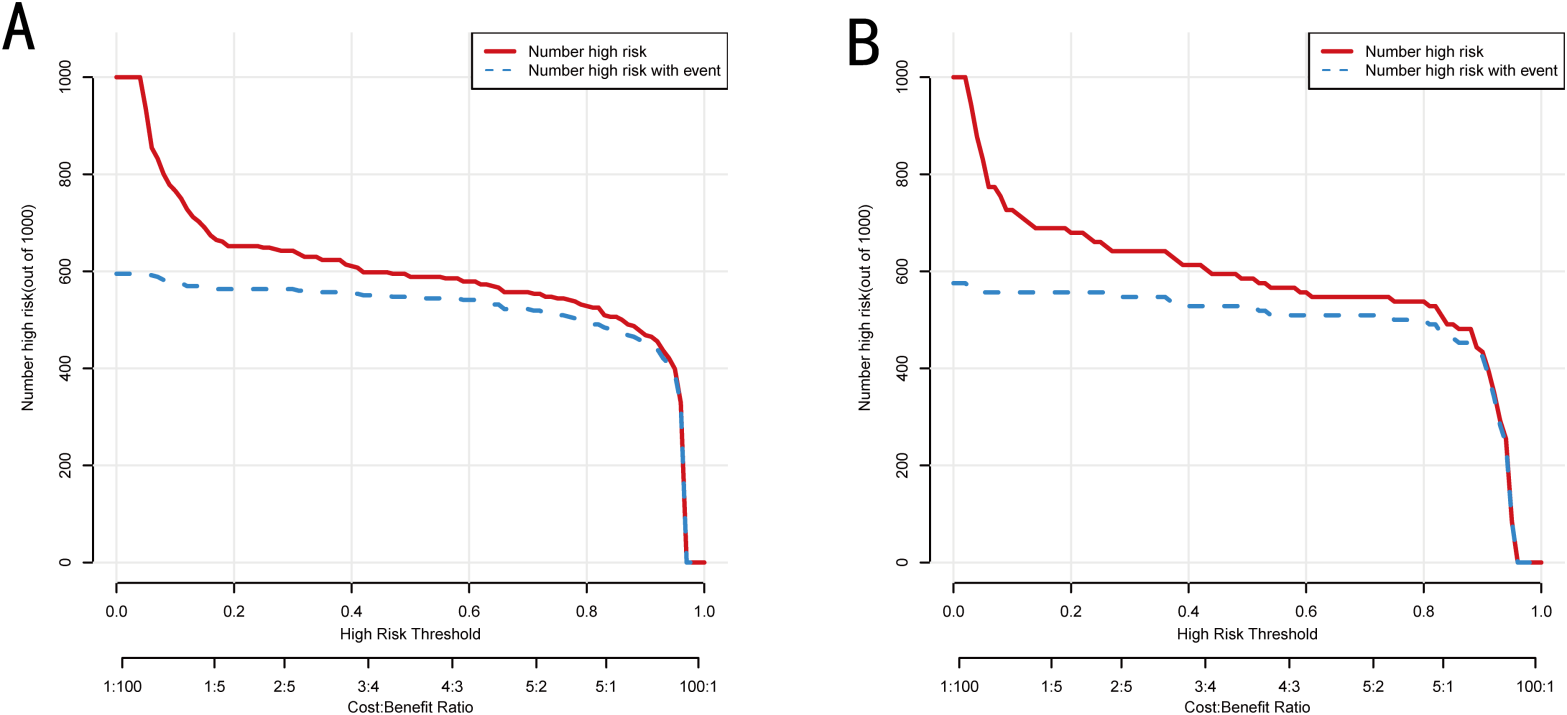
CIC of the nomogram in training set **(A)** and validation set **(B)**.

DCAs showed that the nomogram model offered a greater net benefit than any single predictor, particularly for threshold probabilities within the range of 0.05 to 0.97 in the training set as well as 0.07 to 0.96 in the validation set (**Figure 6**). Furthermore, CIC indicated that the model performed well in predicting high-risk PA patients, with results that closely aligned with actual incidence rates (**Figure 7**).

## 4 Discussion

This study introduces, for the first time, a multidimensional, data-driven nomogram model that leverages clinical characteristics and hematological indicators to accurately differentiate between PA and WT preoperatively. Utilizing LASSO regression and logistic regression analyses, independent predictors—including sex, age, smoking history, bilateral parotid involvement, WBC, and PBG2h—were identified for model construction. The results indicated that the model achieved an AUC value of 0.951 in the training set. The validation set demonstrated consistent performance, with an AUC reaching 0.934, confirming high sensitivity, specificity, and overall accuracy. The results of the calibration curve and the Hosmer-Lemeshow goodness-of-fit test showed that there was a high degree of agreement between the predicted probabilities of the nomogram model and the actual observed frequencies, which confirmed the excellent calibration performance of the model. Moreover, the DCA and CIC further verified that the model has important application value in clinical practice. Compared to FNAC and imaging techniques, this nomogram is a non-invasive, affordable, and easy-to-use diagnostic tool. It is especially useful in places without many resources, as it only uses normal blood tests and medical history. The standardization of hematological indicators and clinical data effectively minimized human bias, enhancing the model’s reliability and reproducibility. This study provides a new perspective for preoperative diagnosis of parotid tumors and offers a reference for developing predictive models for other tumors.

Within this investigation, a higher proportion of males (95.38%) were observed in the WT group compared to the PA group (58.63%), a finding that is consistent with those reported in other studies (22, 26). Considering that smoking serves as a standalone risk factor for WT (27), the gender disparity between PA and WT may be related to smoking, as women in China and Turkey smoke less than those in Western countries (7, 28, 29). Additionally, the WT group’s median age was 58 years, which is higher than the PA group’s median age of 38 years, as reported elsewhere (22, 28, 30, 31).

WT is often characterized by multifocal growth and bilateral parotid involvement, observed in approximately 2.10% to 27% and 3.8% to 30% of cases, respectively (28, 32). This study found a higher prevalence of bilateral parotid involvement in the WT group (10.40% vs. 0.40%) compared to the PA group, supporting bilateral involvement as a key distinguishing feature between PA and WT. While some studies suggest a link between bilateral involvement and Epstein-Barr virus (EBV) infection (33, 34), others have failed to detect EBV-DNA in WT cells, necessitating further investigation into its pathogenesis (35, 36).

According to current consensus, inflammatory reactions are closely linked to the development of WT (37). Accumulating evidence suggests that chronic inflammation is not only a hallmark of WT but also a key driver in its pathogenesis. Recent studies (37, 38) have highlighted that chronic inflammation plays a pivotal role in WT pathogenesis through multiple mechanisms: (1) persistent inflammatory microenvironment promotes mitochondrial dysfunction in epithelial cells leading to oncocytic transformation; (2) senescence-associated secretory phenotype (SASP) from aged cells perpetuates inflammatory responses; and (3) lymphoid stroma in WT actively maintains chronic inflammation through germinal center formation.

Chronic inflammation is also closely related to aging, and the risk of WT increases significantly with age, with an estimated 9.4% increase in incidence per additional year of age (OR = 1.094) (39). Aging cells secrete inflammatory factors (such as IL-1α and IL-6) through SASP, which intensifies local inflammatory response and promotes the proliferation of tumor cells by activating NF-κB and STAT3 pathways. In addition, mitochondrial dysfunction related to aging may further amplify inflammatory signals and form a vicious circle (37). These findings suggest that WT should be classified as an age-related disease, where aging-associated inflammation contributes to the initiation and progression of the tumor. Despite WBC levels staying within the normal range, the WT group in this study had considerably higher WBC and inflammatory markers such as NLR, LMR, and SII than the PA group. A persistent low-grade inflammatory condition could be indicated by the rise in WBC. In contrast to PA, which is mainly composed of fibrous tissue and epithelial cells, WT, which are made up of tumor epithelium and lymphoid stroma, is more likely to cause inflammatory reactions. This idea is further supported by WBC being identified as an independent predictor.

Kadletz et al. (9) reported a significant increase in BMI in WT patients (29.1 vs. 26.2, p < 0.0001), as well as a higher prevalence of metabolic syndrome-related comorbidities in WT patients (62.4% vs. 35.2%, p < 0.0001), suggesting obesity as a contributing factor to the development of WT. This is consistent with studies showing that components of metabolic syndrome—such as obesity, hypertension, and hyperglycemia—promote chronic inflammation through adipocytokine secretion and transforming growth factor-beta (TGF-β) pathway activation (40, 41). Gontarz et al. (39) served similar findings. However, due to the interplay between hypertension, overweight/obesity, hyperglycemia, and inflammation, this study compared the differences in metabolic indicators such as hypertension, diabetes history, BMI, and blood glucose levels between PA and WT patients. The WT group exhibited significantly higher rates of hypertension, overweight, fasting blood glucose, and postprandial glucose at 2 hours. LASSO regression analysis identified PBG2h as an independent risk factor for differentiating PA from WT. Based on existing literature, we speculate that obesity-induced increase of inflammatory factors in adipose tissue (such as macrophage, TNF-α and IL-6) and systemic inflammatory markers (such as CRP and NLR) (42) may provide a pro-inflammatory microenvironment for WT and participate in the pathogenesis of WT. It is worth noting that obesity and metabolic syndrome are themselves associated with accelerated biological aging and increased levels of systemic inflammaging markers, which may further exacerbate the age-related inflammatory milieu conducive to WT development. Overweight or obesity may promote fibrosis and inflammation, leading to parotid dysfunction (43). Additionally, abnormal levels of sialic acid, phosphate, and peroxidase activity in obese individuals could contribute to these effects (44). Furthermore, hyperglycemia may enhance the expression of TGF-β2 and its signaling pathways, promoting extracellular matrix protein deposition in the parotid gland, thus reducing salivation (45). Hyperglycemia can also damage the parasympathetic nerves of the parotid gland, leading to vasodilation, decreased salivary flow rate, and increased total protein levels in saliva (46).

Despite the fact that this study obtained rich data sources and set high standards for factor selection to enhance the rigor of the model, there are limitations to this study. There may be a selection bias because the sample of subjects was recruited from only one medical center. The observed variations in hematological parameters may have been due to unnoticed coexisting diseases, differences in laboratory analysis techniques, or lifestyle factors such as smoking, which was not specifically considered. Furthermore, some of the predictors are still uncertain in terms of how they may be associated with the pathogenesis of PA and WT. Additionally, individuals who received conservative observation treatment may not have been included in the model, as it is primarily based on pathology data. This study focused only on pleomorphic adenoma (PA) and Warthin tumor (WT), the two most common benign parotid tumors. However, salivary gland tumors are a very heterogeneous group of lesions with many different histological types. For example, oncocytoma, a benign tumor often seen in elderly patients, can also occur bilaterally in the parotid gland, similar to WT. Risk factors by tumor type and demographics may vary in different regions, and therefore, it may be necessary to fine-tune the model to the specific local environment.

## 5 Conclusions

In conclusion, the six-variable diagnostic model to differentiate PA from WT, based on sex, age, smoking history, bilateral parotid involvement, WBC, and PBG2h, exhibited good predictive performance in both training and validation datasets, as well as strong calibration and clinical applicability. This model constitutes a non-invasive, economical means of early diagnostic aid for appropriate differentiation between PA and WT.

## Data Availability

The raw data supporting the conclusions of this article will be made available by the authors, without undue reservation.
